# Industrial Symbiosis for Sustainable Management of Meat Waste: The Case of Śmiłowo Eco-Industrial Park, Poland

**DOI:** 10.3390/ijerph20065162

**Published:** 2023-03-15

**Authors:** Zygmunt Kowalski, Joanna Kulczycka, Agnieszka Makara, Giovanni Mondello, Roberta Salomone

**Affiliations:** 1 Mineral and Energy Economy Research Institute Polish Academy of Sciences, Wybickiego 7a, 31-261 Cracow, Poland; 2 Faculty of Management, AGH University of Science and Technology, Gramatyka 10, 30-067 Cracow, Poland; kulczycka@meeri.pl; 3 Faculty of Chemical Engineering and Technology, Cracow University of Technology, Warszawska 24, 31-155 Cracow, Poland; agnieszka.makara@pk.edu.pl; 4 Sustainability Lab, Department of Economics, University of Messina, Via dei Verdi, 75-98122 Messina, Italy; giovanni.mondello@unime.it (G.M.); roberta.salomone@unime.it (R.S.)

**Keywords:** sustainability development, eco-park, meat waste, resource efficiency, closed-loop system

## Abstract

This study presents the developing process of the Śmiłowo Eco-Park, located in the Noteć valley region (Poland), is a part of the biggest Polish agri-food consortium, from its initial small waste management company to its final structure as an eco-industrial park using industrial symbiosis methods. The industrial symbiosis applied in the Eco-park promotes a business model which covers the whole life cycle of the products starting from the plant growing by animal feed preparation, livestock breeding, meat preparations, meat-bone meal production from animal waste, and the use of pig slurry as a fertilizer. The Eco-park model is presented in the form of a system of connected stream flows of materials and energy covering the full lifecycle of products, from cereal cultivation, through the production of industrial feed, and poultry and pig breeding for the production of meat products. The solutions used include the prevention of environmental pollution through the modernization of existing processes, implementation of new technologies, reduction of waste and its reuse, recycling, and recovery of materials and energy, the substitution of raw materials with waste, and thermal treatment of waste and its use as biofuel. This case study allows for analyses of the organizational and technical key strategic activities which enable waste, including hazardous waste, to be transformed into valuable materials and energy. These activities have modified the system of material and energy flows through the value chain to realize the goal of allowing profitable management of waste according to circular economy methods and also indicates methods of supporting modifications of supply chains in terms of implementation of the industrial symbiosis business model according to its relationship with sustainable development, cleaner production, and circular economy models. EIP Śmiłowo annually utilizes 300,000 t meat waste, produces 110,000 t meat bone meal biofuel, uses 120,000 t of pig manure as fertilizers, produces 460,000 GJ bioenergy, eliminates 92,000 t CO_2_ emissions.

## 1. Introduction

Industrial symbiosis (IS) connects industry sectors that typically work separately into collective company teams, increasing the possibilities of their development thanks to the effective use of common flows of energy and materials. Primary to IS is cooperation and synergy resulting from the close location of cooperating companies [1]. This topic has been widely discussed since the first seminal contribution of Frosch and Gallopoulos [2], and nowadays, it is recognized for its role in promoting sustainability [3], as well as its connection with other concepts, such as the circular economy (CE), cleaner production (CP), and sustainable development (SD).

CE assumes that it is a continuous development cycle that preserves and enriches natural capital, optimizes raw material profits, and minimizes systemic risk through appropriate management of non-renewable and renewable material streams. The model of such economic growth is characterized by designed renewability and reproducibility, and its goal is to constantly maintain the highest value and utility of products, components, and materials in separate cycles: biological and technical [1,4,5,6,7].

With the rapid development of CE discourse in recent years [4], a growing interest in IS is observed in industrial and policy applications. IS allows cooperation among companies that traditionally be separated, in the sharing of resources, to increase sustainability with environmental, economic, and social benefits. The symbioses between industry and the surrounding community also have great potential for development with numerous advantages for both parties [3]. IS being a precursor concept with respect to the European vision of the CE model, it is nowadays considered as the meso-level perspective of circularity. IS implemented at the microeconomic level, in manufacturing companies allows for the acceptance of the circular economy program assumes that the company pursues various strategies to improve the production system and works with other companies in the supply chain to achieve a more economically efficient circular cycle [5,6]. In addition, IS is an approach for putting a CE into practice by miming nature’s system in an industrial environment [7] and by improving resource efficiency through inter-firm cooperation [8]. IS can be also treated as a systemic and operational tool used in many sectors to transition toward a CE [9].

The scope of IS is based on the idea of using broadly defined underutilized resources, which results from longer productive use [5]. The IS approach decreases the consumption of primary materials, proposes new reuse and recycling activities of waste, and prevents waste generation. IS allows for increasing profitability and maximizing the amounts of products that can be obtained from resources. This allows the implementation of a CE and green development through symbiotic activities between organizations [10].

IS focuses on the ecological, social, and economic CE influences due to possible benefits in these areas [5]. IS goal is the achievement of environmental, economic, and social objectives. Finding solutions that limit resource consumption and greenhouse gas emissions is essential to ensure sustainable economic growth. The model of such economic growth is characterized by designed renewability and reproducibility, and its goal is to constantly maintain the highest value and utility of products, components, and materials in separate cycles: biological and technical.

Acceptance of the circular economy program assumes that the company pursues various strategies to improve the production system and works with other companies in the supply chain to achieve a more economically efficient circular cycle [8,9,10]. As an accelerator of innovation, IS results in mutually beneficial inter-organizational partnerships leading to waste minimization. CP is considered a basic strategy for CE and SD by implementing cleaner productions, technologies, and services to prevent environmental pollution and prevent the use of non-renewable materials in all phases of the product life cycle. CP technologies include processes such as reduction at the source, in-process, on-site/off-site recycling, and the replacement of primary resources by waste [11]. CP allows the constant action predicted by the United Nations Environment Program UNEP of a complex preventive ecological strategy policy to decrease environmental hazards [12]. IS could also be used in terms of the life cycle or supply chain [13,14] or by a collaboration of companies, shaping the IS model [15].

Bocken et al. [16] refer to IS as an SD model that creates benefits from waste. Additionally, IS is considered to be an important tool for SD that allows the implementation of the UNEP’s Sustainable Development Goals (SDGs) [10,17]. Indeed, IS assumed possibilities for existing companies to increase their competitiveness by minimizing material consumption. Other benefits resulted from decreasing demands for raw materials and decreased waste release. IS supports regional development by developing new innovative productions utilizing unused material/energy streams [18,19,20]. These economic, ecological, and social profits of symbiotic synergies resulted from a decrease in raw material consumption and waste landfilling costs, the generation of revenue from waste, and the minimization of CO_2_ emissions [3].

CE activities at the meso-economic (sectoral) level relate mainly to the production side, including the development of eco-industrial parks, industrial symbiosis networks, as well as other related production networks. IS also allows for reaching eco-efficiency and regional competitiveness objectives [21], strengthening local economies through improved use of material and energy streams. Cooperation in IS decreases the consumption of primary materials and landfill waste, closing the material loop, which is the most important goal of CE eco-innovative solutions [11]. Such cooperation can be put into practice by implementing eco-industrial parks (EIPs) as a solution where companies can use the value chain to decrease waste and increase the value and economy of scale in used technologies.

Industrial eco-parks [1] are one of the oldest forms of the circular economy. Eco-parks are industrial zones that promote collaborations between businesses and local communities, generating environmental, social, and economic benefits. Eco-parks that foster exchanges of materials, water, energy, and information between interdependent businesses operating complementary activities, along the lines of natural symbioses, are described as examples of industrial symbiosis. For today’s organizations, applying circular models can yield enormous benefits in several business-critical areas. However, eco-industrial parks are almost always confined to the space of the park, and indeed industrial symbiosis can be triggered without the demand for geographic proximity between the participants. Proximity is a factor that facilitates the creation of synergies and reduces waste transportation costs [10,15].

Worldwide, the number of industrial eco-parks is growing rapidly: up from 245 in 2001 to 438 in 2020. They are found particularly in Asia (China, Japan, and South Korea) and Europe. Eco-park creation reduces the environmental impacts of industrial activities by promoting synergies, as well as the economic benefits associated with shared resource management. Creating industrial symbiosis, including the choice of technologies, depends on the types and quantities of materials and resources available in and around the parks [7]. Every situation is different and must be studied in detail beforehand to identify the potential that can be exploited. Analyses of technologies, infrastructure, governance systems, and business models in three types of activity found at industrial eco-parks: energy, water, and materials and waste showed that 35% of eco-parks have installed renewable energy sources; almost 50% have adopted efficient water practices to optimize water use and recuperation; and 52% have set up an industrial symbiosis program to recover waste and material flows within the park, particularly to generate heat. Barriers to eco-park development in terms of exchanges between industrial companies are a lack of information about resources and materials available for recovery and a lack of trust between actors when it comes to sharing confidential data and committing to joint investments. However, it recommends setting up digital platforms to identify reserves of materials and waste and their sources, as at the Norrkoping industrial park in Sweden. Local and national authorities should establish tax incentives, create financing mechanisms and make it easier for parks to share the lessons learned. Park operators should adopt the World Bank and UNIDO industrial eco-park toolbox, which provides detailed guidance on rolling out circular economy principles for projects of this type [6,7].

Since 1996 case of the ecological and industrial park Kalundborg in Denmark is presented as one of the most successful cases of the first implementation of industrial symbiosis and the most cited in the literature. Kalundborg saves the city EUR 24 million annually, as well as eliminating 635,000 tons of CO_2_, 3.6 million m^3^ of water, 100 GWh of energy, and 87,000 tons of solid materials. The Kalundborg Eco-Industrial Park demonstrates how resource sharing among industrial actors in the same locality can be beneficial both ecologically and financially. Stories about circularity and industrial symbiosis such as Kalundborg’s help organizations better position themselves to capitalize on a more sustainable and profitable future [1,7]. This is an example of EIPs founded on the IS basis, the inter-firm cooperation can be reached across companies operating in the same sector or among different sectors [7,22].

Industrial symbiosis is a tangible solution enabling circularity within and across value chains. IS concept is defined as “place-based exchanges among different entities that yield a collective benefit greater than the sum of individual benefits that could be achieved by acting alone”. In practice, this is commonly set up in a way where one company’s output can become another company’s input, allowing for simultaneous improvements in resource efficiency and financial benefits [1,3,7].

In addition to these objectives, the article [3] also intends to provide a quantitative view of industrial symbiosis. Although there were some studies with quantitative evaluations, these were related to articles published until 2012 [3] and are therefore not currently up to date. The lack of some economic data related to IS and EIP case studies may be a barrier to IS development [3,23], particularly when real IS systems are realized, but data related to their implementation is not available.

An example of an ecological industrial park can also be a production complex of companies belonging to the Farmutil concern cooperating with each other in the Śmiłowo area. The exchange of waste, water, energy, and materials between cooperating companies significantly increases their environmental and economic efficiency and has also created other less tangible benefits for these entities, such as improving the image of companies or sharing staff, equipment, and information.

In this context, this study on the origin and development of the Śmiłowo EIP (as part of the Farmutil consortium in Noteć valley, Poland) aims to assess the process of organization of the IS in the area of the meat and agri-food industries, considered as one of the most important sectors among which the principles of a CE should be achieved [24], by which wastes have a high potential to be valorized through IS strategies [25]. Additionally, the meat industry has the biggest ecological problems connected with the generation of hazardous waste, which is a great chance for implementing IS actions in the plants, due to the additive benefits from their products and by-products [26]. For industrial production dealing with hazardous waste, the implementation of IS is still a challenge [27,28]. Furthermore, proper regulations and rules of hazardous waste management will facilitate eco-industrial networks, which could be problematic for many organizations [29,30]. The focus on this specific EIP is relevant considering that results from this study may be useful for companies that want to implement IS in the specific agri-food production segment.

The study goal is to update and expand the IS analysis by focusing on the description of this real case study of Śmiłowo Eco-Park to identify the most important activities that allowed for the successful implementation of an IS model. The work presents analyses of the organizational and technical key strategic activities which enabled waste to be transformed into valuable materials and energy, closing material cycles. In addition, the study discusses the incorporation of the IS approach in the Śmiłowo EIP model in relation to the SD, CP, and CE models.

## 2. Methods

Farmutil is one of the largest and most modern agricultural and food concerns in Poland, with several dozen production plants throughout Poland. The model solutions used and planned for implementation in Farmutil are an example of applying the principles of closed-loop economy in industrial practice, based on the methodology of cleaner technologies, on a microeconomic scale. System analysis covers the full life cycle of products, from obtaining raw materials, through production, and use, to disposal of the used product. The comprehensive nature of the product assessment “from cradle to grave” means that no stage of the product’s existence is omitted. Ultimately, it is assumed that the entire production system of the Farmutil concern will be transformed into a waste-free one using cleaner technologies and circular economy systems [31,32,33].

In this research, a modified case study analysis inspired by the Yin method [34] was applied to understand the significant determination of the most important factors that guided the birth and evolution of the Śmiłowo EIP, using updated and systematized data available from previous research [31,32,33].

The study analyzed a retrospective collection of data covering all the important structural changes to understand the internal and external drivers that guided the transformation of Farmutil from a small waste management company into a large agri-food consortium, as well as the barriers encountered. The exchanges of waste, energy, and materials between cooperating companies have been investigated to determine how these exchanges increase the firm’s environmental and economic performance and create other less tangible benefits for these entities, such as improving the image of companies.

Currently, there are three levels of indicators for measuring circular economy: macro (global, national, regional, city), meso (industrial symbiosis, eco-industrial parks), and micro (single firm, product). A detailed understanding of how to measure progress towards a circular economy and IS development is lacking, especially on a micro level. This is a barrier for both producers who want to provide circular products and services, and for the consumers who want to know how to compare products [35].

Due to this, the evaluation made of the effects that resulted from the development of the Śmiłowo EIP was qualitative. Indeed, the assumption is that the development of the IS network may increase the quantity and the type of resources used, which resulted in increased ecological profits [18]. Usually, the created value chains have developed autonomously and are driven by various industrial initiatives and activities [36]. Following this, the case study method has been carried out following four research steps: (1) case study description, (2) quantification and mapping of the main industrial symbiosis networks, (3) evaluation of the activities in terms of SD, CP, and CE, and (4) analysis of drivers and barriers in the Śmiłowo EIP implementation. The quantification and mapping of the main symbiotic exchanges have the main goal to grasp the characteristics of the network with a focus on the main streams of resource flow between the production and service EIP companies and the main technical and organizational solutions they implemented.

Then, the strategic activities realized in the EIP are identified and analyzed according to their relation with the SD, CP, and CE models due to the prioritization of IS initiatives, which should be based on a detailed understanding of the SD principles [37], also considering that strategic data due to a CE includes not only waste prevention and a decrease in waste but inspires technical, economic, and social innovation in area value chains [33,38,39].

Then, the effects on the sustainability of Śmiłowo EIP development are assessed using a triple button line qualitative estimation of the above-mentioned activities that were implemented in the EIP case study [40,41]. Specific EIP effects considered are as follows:

- Economic effects are evaluated in terms of profitability resulting from higher revenues from realized CE enterprises and collaboration in terms of more productive utilization of material flow streams through companies. In addition, IS leads to a new collaboration and cross-value chain partnership, lower cost, and an improved cost system, with a balance among various scales of business. Innovative products with higher quality and enhanced utility should be the basic drivers of the versatility and elasticity of IS firms [42]. EIP companies should have decreased material and energy consumption, resulting in lower operating costs. This contains the recycling and reuse of materials and increased resource yields by circulating high-quality materials [3].
- Environmental effects refer to waste release quality, their amount, raw materials and energy use, reduction of waste toxicity, and management of by-products and waste. This resulted from the development of new methods of meat waste management and treatment, reuse of recycled products, and bioenergy recovery from waste, which allows for the decrease of operating costs because of using innovative technologies determined as the most accessible techniques that do not result in increased costs [33].
- Social benefits contain changes in consumption models through a socially acceptable use of goods. Higher quality wares resulted in an advantageous influence on human health. Participating and cooperating commerce practices make more economic, ecological, and social profits for consumers and stakeholders.

Finally, a study on drivers and barriers to the implementation of Śmiłowo EIP is performed. Drivers and barriers of IS are identified and analyzed according to the problem area (i.e., physical/technical, regulatory, resources, collaboration) and level (inter- or intra-organizational, company, employee) in which they occur [43,44].

## 3. Results and Discussion

### 3.1. Case Study Presentation

Śmiłowo EIP belongs to the Polish Farmutil agri-food consortium, consisting of 13 firms with 36 production and service installations. The implemented EIP symbiotic actions follow the Farmutil consortium strategy, creating a symbiotic production model including the whole life cycle of obtained products [33,34]. Business models adopted by the involved cooperating firms cover material and energy streams interchange, allowing for the implementation of the system of a progressive value chain [3,45]

**Figure 1:**
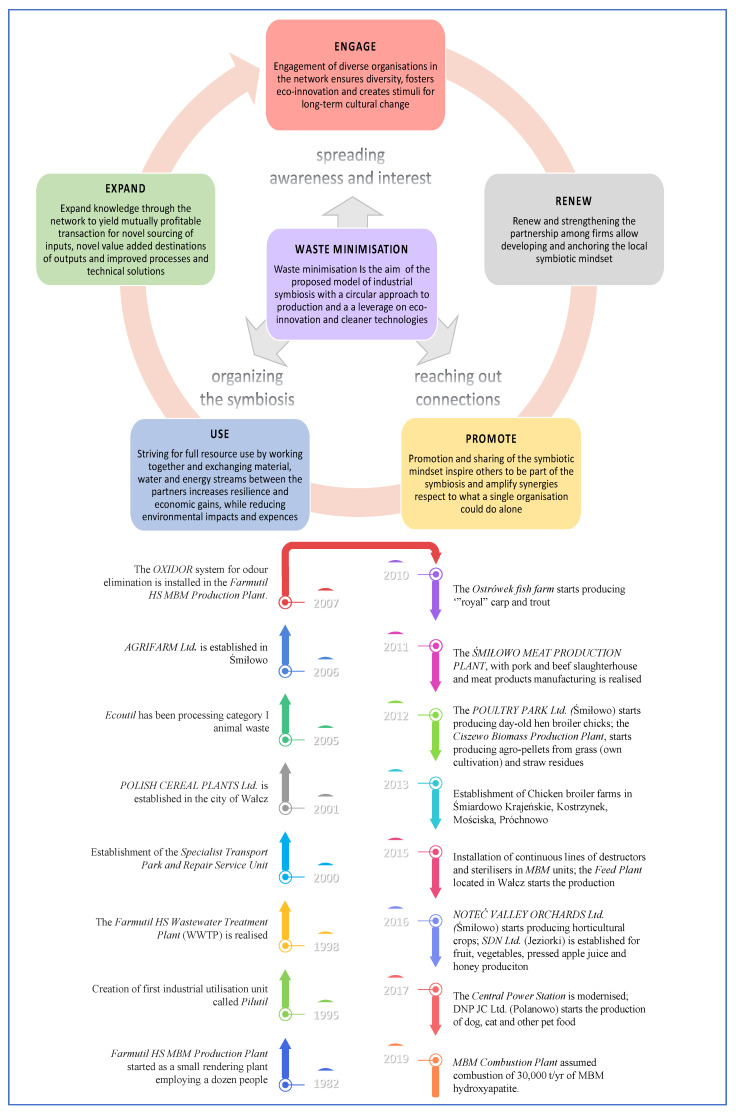
Key actions and main steps for the development of industrial symbiosis in the Śmiłowo Eco-Industrial Park (from 1982 to 2021).

The Farmutil consortium is a driving force of EIP development that implemented a coherent management system of the production complex for guiding strategic development directions, new investments, and their location. Farmutil’s strategic goal is to decrease hazards to the environment through the development of new waste-less and energy-saving technologies using bio-waste for biofuel production [33]. The evolution of the EIP is summarized in Figure 1, accordingly to [33,34,46].

The EIP progressively evolved by implementing three key actions in IS: spreading knowledge and interest in IS, determining and use of the symbiotic network, and developing the symbiosis. The key actions were translated into the following operative steps:

- Engagement of firms in the diverse perspectives and changes connected to IS creates an atmosphere of inclusion and growth. The various organizations collaborated in the network to ensure diversity and foster long-term eco-innovation. These changes require a joint effort from everyone, from leadership to the front lines of the employees.
- Renewing and strengthening the partnership among firms allows for the development of the local symbiotic mindset. Renew also implies how important it is to still recognize and join new participants to develop new chains.
- Inspiration and participation in the way of symbiotic thinking stimulate other firms to be a member of the symbiosis. Partnerships thrive and create synergies when partners see that they can do more together than they could do separately. Achieving this takes a careful understanding not just of how partners work together but also of the unique strengths, risks, and needs of each partner.
- Striving for full resource use through an inter-firm collaboration based on materials and energy exchanges allows high resilience and economic growth, as well as the reduction of environmental impacts, using European Circular Economy Stakeholder Platform ECESP according to different IS models [47].
- Knowledge to expand the business using the value chain to yield many beneficial transactions for a new finding of inputs, value-added final solutions, and enhanced technology and business methods. Business action demands using a wide range of knowledge and experience. The system of business data collection using its own knowledge could be the most important element for effective development.

### 3.2. Quantification and Mapping of the Main IS Exchanges

EIP Śmiłowe consists of 13 firms with 36 production and service installations. The proximity of the EIP firms plays an important role, and most synergies happen within a radius of fewer than 50 km. Before the start of the project, the optimum localization of planned installations should be found, the maximum possibility of shorter transport ways to firms, and the chance of successful implementation should be evaluated.

**Figure 2:**
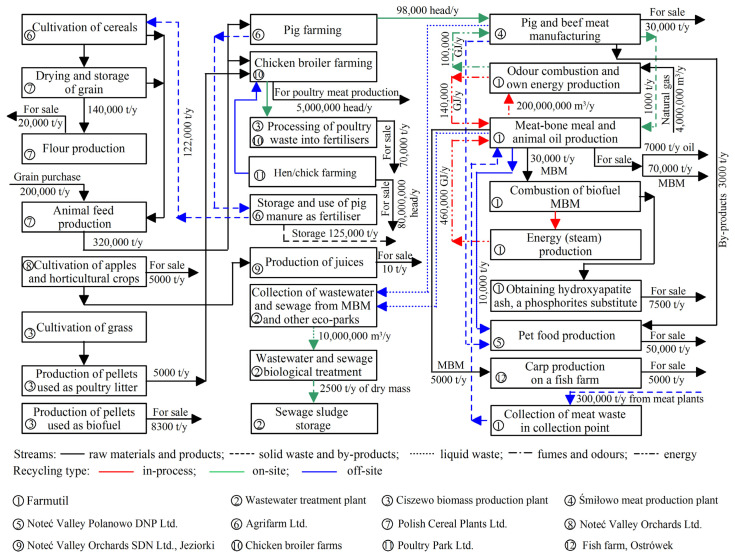
Exchange flows of materials and utilities in the Śmiłowo EIP production units.

MFW analyses allow to presentation worked model of the exchange of stream flows of materials and utilities in the Śmiłowo EIP production units, summarised in Figure 2. In particular, quantities of flow streams of raw materials, products, energy, waste and by-products, liquid and solid waste, fumes, and odors are shown. Additionally, recycling types (in-process, on-site, and off-site and connection between EIP units) were marked. These activities connected with waste management are realized by using proper material flows to achieve the EIP’s objective is most important for the sustainable management of waste. Śmiłowo EIP companies are being placed together, creating a closed-loop system, characteristic of an EIP, using cross-value chain cooperation.

Farmutil Inc. (Śmiłowo, Poland) in Śmiłowo is the biggest EIP company that realized the utilization of meat waste to produce meat bone meal MBM. The obtained category III MBM, as defined according to the European Legislation (EC) No. 1069/2009 and No. 1774/2002 [48,49], is utilized for the production of pet food and fish feed, as well as for the production of biofuel. Two working Farmutil MBM units (Pilutil and Ekoutil), due to the applied technology and apparatus solutions, can be counted among the most modern in the EU. Their capacity allows the utilization of 600,000 t/y meat waste (60% of produced in Poland). Over 100,000 t MBM and 20,000 animal fat (high-calorie biofuel) are annually obtained. Last year, the MBM unit used the new destructors/sterilizers operating in a continuous system, which allowed to increase in the efficiency of MBM production lines and reduced 10% the costs of this production [33]. In Farmutil, the OXIDOR system has been used since 2010 for combustion with natural gas all the gaseous fumes from individual technological operations, and air from production rooms, containing odor and vapors (total quantity—200 million m^3^/y). The implemented thermal methods eliminated odor emission from MBM plants, and are the most effective, although expensive, and therefore rarely used [32,33]. Implementing technology for the elimination of odor emissions, from a strategic point, was extremely important for the company’s image. Its implementation improved relationships with the public. For example, the number of complaints about odor emissions directed by local residents to environmental protection institutions decreased from an average of 200 per year to zero after the full implementation of the OXIDOR system.

In 2019 Farmutil implemented a biofuel MBM combustion project that assumes a combustion of 30,000 t/y of MBM, a production of 460,000 GJ/y of steam, and 7500 t/y of ash containing hydroxyapatite. Steam production based on MBM combustion allowed a former Farmutils coal-fired heating plant to close and eliminate consumption of over 25,000 t/y coal [34,46]. Ashes from the thermal waste utilization of MBM contain hydroxyapatite, which is a high-quality substitute for phosphates used in the production of phosphoric acid and phosphate salts (50,51). A developed capacity of 7500 t/y of ashes. Heat production based on MBM combustion allowed to close off of the former old coal-fired heating plant and eliminate consumption of over 25,000 t/y coal. These resulted in the elimination of such impurities [t/y]: SO_x_–350; NO_x_–52.5; CO–1250; CO_2_–50,000; dust (suspended); benzo(α)pyrene–0.57; heavy metals (∑Cd, Pb, Hg, As, Cr, Cu, Ni, Zn)—approximately 75 and savings on the fee for CO_2_ emissions (60 EUR/t of CO_2_) amounting to 5.5 MEUR/y.

In the presented model there is also a whole range of auxiliary and service installations for all the Eco-park units. Farmutil’s Specialist transport park includes over 2000 trucks and different cars. Owning a huge and very diverse modern car fleet allows for more efficient distribution and sales, and transportation services.

Repair Services Unit, first of all, is necessary to mention its own production and maintenance facilities for the production and transport of equipment. This allows devices to be regenerated and their usage time to be extended, which is one of the important elements recommended for use in CE.

Central Power Station (modernized in last 3 years secures electricity demand for Eco-park production and auxiliary units.

DNP Ltd., Polanowo is a producer of dog, cat, and other pet food. Production of wet pet foods from the premium and super premium segment based on using of non-food meat residues is intensively developed. In this production is used, among others, category III meat-bone meal, thus closing the meat product cycle [34].

Drinking water intakes and production units produce food-quality drinking water for juice production, chicken breeding, meat production, and other purposes. Water is taken from own wells (capacity over 600,000 m^3^/y) located near the Narew river and treated by filtration method [34]. This water is also used to produce natural apple juices made from apples grown in our own orchards. Own production of food-quality water positively influences the quality and costs of products manufactured using it.

The effectiveness of the Śmiłowo EIP is very high due to the production of own raw materials used in EIP companies that produced and sold final products in the market. This also results from the fact that all of the companies are being placed together, creating a closed-loop system, characteristic of an EIP, using cross-value chain cooperation. In particular, activities connected with waste management are realized by using proper material flows to achieve the EIP’s objective, which is the sustainable management of waste.

Table 1 present summarized data in terms of material and energy stream flows exchange per year among the Śmiłowo EIP included data on the types of products manufactured by companies of EIP Śmiłowo, their production volume on an annual basis, indicated amounts of materials used as raw material for own use, disposal and sale, as well as the amount of energy generated and waste processed. EIP Śmiłowo utilizes 300,000 t/y meat waste into the production of 110,000 t/y MBM of biofuel, uses 120,000 t of pig manure as a substitute of artificial fertilizers, produces 460,000 GJ energy by combustion MBM biofuel.

Table 1 also evaluated the income from the sales of main products EIPs Śmiłowo. Total income is estimated to be over EUR 520 million. Detailed data on operation costs in the Farmutil consortium are not available, but some information collected [33,34,50,51] allowed evaluation that EIP’s gross profit could be annually about 20%, i.e., over EUR 100 million.

The Śmiłowo EIP has also obtained the most advantageous service system, auxiliary services, and investment companies working for all of the EIP production plants (Table 2).

### 3.3. Evaluation of the Activities in Terms of SD, CP, and CE

The IS strategies implemented in the EIP have modified the system of material streams through the value chain to manage waste in a more sustainable and beneficial way. Their description and the specific technical and innovative solutions implemented are specified in Table 3, which reports the results related to the evaluation of the EIP in relation to the SD, CP, and CE models, based on the four identified strategic activities. These key activities allowed the implementation of the IS model, not only by eco-friendly CP and eco-efficient solutions but also at the organizational and social levels. Such solutions refer to the implementation of the value chain of stream flows to facilitate the recycling and reuse of energy and materials from treated meat waste, the complex use of bioenergy, and the decrease in emissions.

Despite different types, CE strategies can be grouped according to their attempt to preserve functions, products, components, materials, or embodied energy; additionally, indicators can measure the linear economy as a reference scenario. The measurement scope shows how indicators account for technological cycles with or without a Life Cycle Thinking (LCT) approach; or their effects on environmental, social, or economic dimensions. However, none of the available indicators can assess the preservation of functions instead of products, with strategies such as sharing platforms, schemes for product redundancy, or multi-functionality. It is suggested that a set of indicators should be used to assess CE instead of a single indicator [39].

Thus, in order to assess the EIP system based on SD, CP, and CE models, four types of activity are taken into account: (a) production for sale and own use, (b) bioenergy production and recycling, (c) waste recycling and reuse, and (d) service and auxiliary.

The four types of activity selected are then analyzed in terms of related qualitative economic, environmental, and social effects, with the results being shown in Table 4. The EIP’s economic benefits could be mostly obtained due to higher incomes from implemented CE solutions and decreasing production costs, resulting in more effective cooperation between EIP firms. IS provides a novel type of collaboration and participation in the cross-value chain and decreases operating costs, which balances the scales of business. New high-quality and increased functionality products are the basic factors of the company’s SD based on IS principles involving the use of novel types of businesses serving as a part of the production and remanufacturing of products, the repair and maintenance of equipment, and the specialized knowledge. The proper location of the newly built units complements the use of existing routes for a more effective way of improving traditional logistics.

Environmental benefits resulted from the lower material and energy consumption figures. This complies with the reuse of secondary materials recovered and optimizes resource use by the circulation of high-utility materials. The environmental effects also include a decrease in hazards to the environment, especially in terms of waste disposal and emission of greenhouse gas GHG. The innovative technologies used for the recycling and reuse of treated wastes and the production of bioenergy resulted in a reduction in the use of non-renewable resources and fuel. Animal and biomass waste recycling/reuse keep waste out of landfills. The network of waste collection points guarantees the continuity of waste supply for processing. The production and use of their own disinfection agents is another exemplification of beneficial packaging recycling/reuse.

Social benefits provide changes in consumption models resulting from socially reliable consumption. High-quality goods have a beneficial impact on human health. Sharing cooperative business experiences results in increased social profits for consumers. It was also stated that distribution influences differ according to the wealth of consumers. Śmiłowo EIP’s location in a region with existing unemployment results in the formation of new jobs.

The IS formation promotes sustainable growth and the development of companies’ business operations, generating eco-innovation activities to introduce resource efficiency and socio-economic outcomes.

### 3.4. Analysis of Drivers and Barriers in Śmiłowo EIP Implementation

The type of drivers and barriers analyzed in the Śmiłowo EIP case and the level at which they occur are shown in Table 5. Typically, drivers and barriers that influence IS creation occur at the inter-/intra-organisational, company, and employee levels [44,52]. Considering all EIP companies are parts of Farmutil’s consortium and unique management system, the inter-organizational level was not included, as it did not cause relevant drivers/barriers.

The diversity of companies belonging to Śmiłowo EIP is an important driver of complex business development. Nevertheless, the most important driver for EIP is a comprehensive strategy of all Farmutil consortiums developed on a consortium intra-organizational level which results in direct technical and business interaction among the EIP’s members. This also makes the partnership with the community and local authorities and between neighboring companies easier.

The IS network improves business, while the technical processes are developed within an accepted consortium strategy. EIPs possess structures that facilitate collaboration, promote the local symbiotic mindset, and inspire firms to be part of the IS model. These also involve a variety of participants in the value chain supporting sustainable development and innovative activities. The provision of financing mechanisms to pay for IS projects is a key driver for successful EIP implementation.

Among IS development, an important barrier is identified in the scarcity of specific knowledge and awareness of the IS principles and CE in firms and of methods/tools for their implementation. Other problems are connected to the estimation of financing viability, as well as to the lack of project business case studies and steering mechanisms for IS development. Some barriers lie in appropriate legal requirements and government intervention and in the lack of resources. Cooperation and networking are costly, which results in corporate inertia. Another problem is the missing demand from customers because the IS implementation purpose is not understood by clients.

Drivers and barriers can be influenced due to the impact of funds, time, or skills; however, it is possible for their replacement by a reduction of production costs or new benefits by improved use of the material flows. The implementation of IS goals is sometimes decreased by barriers connected with the fact that IS has been treated as a low priority and not as an important part of production processes.

Securing the support of key decision-makers helps strengthen funding applications and increases the likelihood of leveraging funding and other support. An increase in innovative firms’ outreach affords more flexibility and the ability to more easily attend to IS creation. These actions represent proactive approaches to increasing awareness of and participation in SD initiatives, as well as exploring firms’ needs while also reducing resource inputs.

Cooperation is another barrier related to issues of trust between companies resulting from their production capacity and organization.

The results presented allow the identification of the most important goals achieved during the EIP development. The first one is the increase of cereal crop production to use more of their own grain for animal feed production. This required an increase in the arable land area and storage capacity in grain elevators. The second one is innovative pet food production methods to use as much of the meat by-products as possible in a profitable manner. The third purpose is to maximize the use of waste-less technologies in the whole EIP by the implementation of new methods for waste utilization and the production of as much bioenergy as possible from MBM. The goal of further development of the Śmiłowo EIP is implementation into the industrial practice of a closed-loop production system to create more economic, ecological, and social profits for all EIP participants.

## 4. Conclusions

The production complex of companies belonging to the Farmutil concern cooperating with each other in the Śmiłowo area is an example of implemented ecological industrial park. The exchange of waste, water, energy, and materials between cooperating companies significantly increases their environmental and economic efficiency and has also created other less tangible benefits for these entities, such as improving the image of companies or sharing staff, equipment, and information.

The presented EIP model is a system covering the full lifecycle of products, from cereal cultivation, through industrial feed production to poultry and pig breeding for meat products. The system also includes the use of meat waste, for the production of meat and bone meal, and waste from pig and poultry farming for fertilizing purposes. Thus, the production cycle is practically performed from the “cradle to the grave”. The development of the Śmiłowo EIP was assessed in terms of the organization and transformation of the IS in the biggest Poland agri-food consortium Farmutil. It focused on the use of new key strategic CE activities for this successful implementation of the EIP model, including the SD, CP, and CE methods, which were qualitatively assessed.

The ecological benefits of the Śmiłowo eco-park result primarily from the reduction of pollutant emissions to the environment because of the implementation of pro-ecological systems, which also results in the reduction of operating costs of the plants because of the implementation of pro-ecological systems. The cooperative business experiences created more economic, ecological, and social profits for all EIP participants who mostly care about partnerships around the mutual use of resources, organizational structures, and access to knowledge. An additional benefit for companies is that IS strengthens their environmental profiles, which can be an important advantage in the market aspects related to production processes and consumption. The social benefits result from changes in consumption models by more responsible use of goods and services.

In the analysis of the EIP Śmiłowo case study, it was stated that basic benefits from the IS cooperation and realization of economic and environmental goals to obtain the higher value of the replacement of by-products and/or waste, due to IS was treated as a methodology that allows for achieving business and ecological success of the individual company, realizing assumptions of industrial symbiosis.

Revenues from the sales of EIP Śmiłowo products are estimated to be over EUR 520 million and gross profit could be over EUR 100 million.

Further studies should apply to the need for a quantitative evaluation of the ecological, economic, and social benefits of the EIP and IS models in general.

## Figures and Tables

**Table 1:** Material and utility stream flows exchanged annually among the Śmiłowo EIP.

Material/Utility	Stream Flows * [t/y]					
	Total	Own Use		For Sale		Disposal
		Amount	Use/Off Process Recycling for	Amount	Income ** [EUR M/y]	
Pig manure	247,000	122,000	Fertilization			125,000
Hazardous meat waste	300,000		MBM production			
Sewage sludge	12,000	12,000	Fertilization			
Meat bone meal	110,000	10,000	Pet food production	70,000	32.11	
Meat bone meal Energy [GJ] produced from meat bone meal	460,000	30,000 460,000	MBM incineration and Energy production for EIP companies			
Animal oil	7000			7000	5.78	
Meat by-product		3000	Pet food production			
Dried grain	20,000		Flour production			
Flour	20,000			20,000	16.0	
Dried grain		120,000	Animal feed production			
Purchased grain	200,000		Animal feed production			
Animal feed	320,000	320,000	Pig and poultry farming			
Pigs	98,000	98,000	Pig meat manufacturing			
Pig and beef meat products				30,000	185.57	
Pete foods				50,000	123.73	
Hydroxyapatite ash				7500	0.87	
Royal carp				5000	41.14	
Day-old hen broiler chicks [pcs]				90 million	55.67	
Chicken broilers				5,000,000	6.5	
Chicken manure fertilizer				70,000	50.51	
Apples				5000	0.41	
Grass pellets	13,300	5000	Poultry litter	8300	3.3	
Energy (steam) [GJ]	240,000	700,000	MBM production and all EIP companies			
Sewage and wastewater [m^3^]	10,000,000	10,000,000	Biological treatment			
Food-grade water [m^3^]	600,000	600,000	All EIP companies			
Odor [m^3^]	200,000,000	200,000,000	Elimination by thermal treatment			
CO_2_			Elimination of emission			
Total income					521.59	

* Market price 2023; ** According to Figure 1.

**Table 2:** Companies providing services in the Śmiłowo EIP also for all the Farmutil consortium.

EIP Units	Service Description
Central Power Station	Electricity supply for the EIP companies
Specialist Transport Park	Meat products and waste transport
Repair Service Unit	Repair and maintenance of transport and production equipment
Investa Investment Service Centre Ltd.	Design and realization of investment with supervision
Investbud Ltd.	Investment realization; design and building of objects, production units, and other investments
Auto Building Ltd.	Container manufacturing
Warehouse for Occupational Health and Safety (OHS) goods	Wholesale and storage of OHS devices
Investa Aero Service	Helicopter and aircraft airport and transport
Biochemik	Research, chemical, and microbiological analyses using certified methods
Weekly News	Information about the EIP and its promotion

**Table 3:** Strategic activities realized in Śmiłowo EIP.

Scheme	Description Based on SD, CP, and CE Models	Company; Belonging Plants/Farms	Production/Service Type
Production for sale and own use	Biomass production. Production of high-quality feed for own pig and poultry farming. High-tech production development using locations in the Nature 2000 area. Development of own poultry and pig farms using modern breeding technology. Development of feed and flour production. Continuous development of farmland area for profitable production of own crops and high-quality feeds. Production of fruit and vegetables, natural juices, and honey. Making the water flow chain shorter with fewer stations.	Meat production plant Śmiłowo.	Pork and beef slaughterhouse and manufacturing plant, producing 100 t/day of meat products. Meat products are produced according to well-proven and very high-quality recipes developed 40 years ago.
		Farmutil Inc., Śmiłowo, Poland; MBM plants: Pilutil and Ekoutil, Śmiłowo.	The biggest EIP company that processes meat waste to produce 100,000 t/y MBM in the most innovative European plants. In addition to the MBM, 20,000 t/y of animal fat is produced as a by-product.
		Biomass production plant, Ciszewo	Production of 8300 t/y agri-pellets from 18,000 t/y of grass (obtained in 5000 ha of cultivated area) and straw residues. The agri-pellet biofuel (12 GJ/t calorific value) substitutes natural energy resources. Additionally, pellets are used as bedding in poultry farming.
		Noteć Valley Polanowo DNP Ltd., Polanowo, Poland	Manufacturing of pet foods, based on inedible meat residues and by-products. DNP uses also category III MBM (off-site recycling). The DNP contributes to job opportunities in an area of high unemployment.
		Agrifarm Ltd., Śmiłowo; Agricultural Plants: a/Śmiłowo, b/Zelgniewo, c/Mościska, d/Zacharzyn, e/Miłostowo, f/Komorzewo, g/Przytoczna	Grain cultivation on 4208 ha. Pig manure is applied to fertilize 4100 ha of land with a standard dose of 44 t/ha, using tankers equipped with splash plates for broad-spreading. This is the cheapest method of applying manure used in Europe. Quantity of pig breeding (heads); Miłostowo (32,000), Komorzewo (16,000), Śmiłowo (1600 sows), and Przytoczna (4000).
		Polish Cereal Plants Ltd., Wałcz, Poland;	Production of feedstuff for livestock, capacity 500,000 t/y. Production of feed (320,000 t/y) totally meets Farmutil’s needs for feedstuffs to use in poultry and pig farming. The innovative unit used modern technology and equipment, characterized by short transport distances and a cascading system of materials flows. Products meet the highest quality and safety standards. The feeds are prepared on the basis of the most up-to-date nutritional knowledge. Their own farms allow the testing of nutritional solutions before introducing them to the market.
		Chicken Broiler Farms: a/Śmiardowo Krajeńskie, b/Kostrzynek, c/Mościska Agricultural Plants: a/Próchnowo, b/Brzeżno	Rapeseed, wheat, and triticale have been cultivated in Brzeźno on 2057 ha and in Próchnowo on 1500 ha. The farms are involved in the breeding of 5 million broiler chickens per year. Broiler chickens are typically white and are specifically farmed to produce meat for consumers. They are fed with top-quality fodder.
		Fisheries Farm, Ostrówek	Breeding of highest quality carps and trout in 14 ponds (area of 424 ha), producing 450 t/y of fish using MBM as fish feed. Localization in ecological park Natura 2000, provided clean water and air and enabled the production of the highest quality products.
		Noteć Valley Orchards Ltd., Śmiłowo, Poland	Cultivation of apples, cherries, pears, and plums on 150 ha and a bee apiary (400 hives of bees). The modern production of fresh, tasty, and healthy high-quality fruits in full correlation with European Union EU environmental regulations.
		SDN Ltd., Jeziorki, Poland	Processing, storage, and sale of fruits, vegetables, apple juice, and honey from Noteć Valley Orchards.
		Poultry Park Ltd., Śmiłowo, Poland	Production of 90 million/y one-day-old broiler chicks from the highest quality feedstuffs. Leading Polish producer using global-level technology.
Bio-energy production and recovery	Bio-energy use and recovery Substitution of coal with biofuel.	Farmutil Inc., Śmiłowo; MBM Combustion Plant, Śmiłowo.	Incineration 30,000 t/y MBM. Production of steam from bioenergy. Energy recovery and in-process heat recycling (for MBM production).
Waste management and processing	Recycling and reuse of processed meat waste. Development of innovative meat waste utilization technologies. Network of collection units.	Take-back waste plants: a/Prusice, b/Węgry, c/Miszewo Wielkie, d/Śmiłowo	High-quality recycling/upcycling collection points with a capacity of 600,000 t/y. Optimum logistic collection of animal and food waste, used for meat bone meal production.
		Wastewater biological treatment plant WWTP, Śmiłowo	A modern WWTP with a high technical level. Obtained sewage sludge is entirely used as fertilizer. Treatment of 10,000,000 m^3^/y of wastewater from MBM and EIP units.
Service and auxiliary activities	Development of own central modern laboratory and research service. Centralized distribution, sales, and transport services. Logistics/infrastructure facilities. Technical service for all EIP companies. Centralized electricity supply and service. Upgrading, maintenance, and repair of equipment and products. Centralized design, investment realization, supervision, and consultancy. Production of disinfecting agents and their application. Maintenance and services of airport, aircraft, and air transport services. Storage and sale of OHS equipment. EIP information and promotion.	Farmutil, Śmiłowo Specialist transport park	Transport services across the EIP: over 2000 trucks and modern vehicles with self-loading and material cooling systems. The transport of the EIP materials is facilitated by the optimal location of the meat collection points and proper distribution of products to the chain of 350 meat stores.
		Polish Cereal Plants Ltd., Wałcz, Poland; Grain Elevator: a/Piła, b/Wałcz, c/Strzelce Krajeńskie d/Drying and storage complex Śmiłowo.	Drying, transportation, purchase, and storage of cereals and rapeseed.
		Noteć Valley Orchards SDN Ltd., Jeziorki, Poland Food-quality water intake and production plant	Provides EIP with drinking water (2000 m^3^/day) treated by the filtration method. The use of food quality and cheap water resulted in the highest quality and lower operating costs. The water is used in the manufacture of poultry and meat products, as well as in obtaining natural juices.
		Service and Research Laboratory Biochemik Ltd., Śmiłowo, Poland	Studies and tests in terms of chemistry, microbiology, sampling, environment, and calibration.
		Auto Building Ltd., Wałcz, Poland	Manufactures containers, trailers, and semi-trailers, as well as other steel structures.
		Investa Investment Service Centre Ltd., Piła; a/Investa Chem Production Plant, Łowicz Wałecki; b/Warehouse for OHS, Śmiłowo, Poland Investa Aero Service LTD., Śmiłowo, Poland	Investment realization, supervision, consultancy, and construction reviews. Production of disinfectants and washing-disinfecting agents. Warehouse of OHS devices. Airport and maintenance services of 5 helicopters and 3 aircraft.
		Investbud Ltd., Piła, Poland.	Investment projects and the realization of industrial units and other buildings.
		Weekly News, Ltd., Piła, Poland	Promotion/information on the Śmiłowo EIP and its companies.

**Table 4:** Qualitative triple-button sustainability analysis of the strategic activities realized in Śmiłowo EIP.

Activities	Triple Button-Line Sustainability Benefits		
	Economic	Environmental	Social
Production for sale and own use	Ensure a strong position in the market. Develop pet food production. Modularity design products with newer features/functionalities. Longer product lifetime. Supply chains become shorter with improved traceability of products and related transactions. Higher materials efficiency. Reduced MBM production costs. Sale of hydroxyapatite ash. Pellets are used as high-quality bedding. System for effective insemination of sows Lower feed/flour production costs due to use of own crops. Decrease pig/poultry farming costs due to the use of their own feeds. Net fertilizer effects due to the use of pig manure. Production of own food-quality water. Higher effectiveness of crop storage and purchasing. More effective cooperation and exchange.	Decrease environmental impact due to reduced release of waste, odor, and GHG emissions. High-quality technologies for recycling/reuse of processed wastes. Manufacturing of bioenergy resulting in decreased consumption of non-renewable fuel resources Recycling of meat waste and non-food meat waste. Take-back structure and collection points guarantee the continuous flow of waste for processing. Own production of disinfection agents results in more effective package recycling and reuse. Proper location of new installations reduces the environmental impacts due to shorter transport routes and complemented use of existing logistics and infrastructure facilities.	Job proposals, especially for qualified workers. Improve relationship with the public due to odor elimination. Certificate for good quality products. Reduce damage to human health. Creation of consumption models for consumers to decrease the use of goods. Better traceability of products.
Energy production and recovery	Profitable production of bioenergy. Low energy cost. Higher efficiency of energy recovery. Reduce costs for heat sharing between companies. Reuse of processed hay/straw as biofuel.	Bioenergy production. Removal of coal use in energy manufacture. Waste-free energy production. Energy in-/off-process recycling and recovery.	Creation of consumption of products using low energy.
Waste management and treatment	Lower wastewater treatment costs. Effective take-back meat waste scheme. Reuse of processed waste.	Recycling and reuse of animal waste and by-products. Wastewater take-back collection systems. Waste-less technologies.	Improve companies’ image in society. Reduce damage to human health.
Service and auxiliary activities	Lower costs of laboratory services. Lower transport costs. Shorter electricity supply chain. Lower electricity costs. Lower investment, repair, and maintenance costs. Lower promotion costs.	Reduce environmental impacts of meat transport and waste collection. Reduce material use.	Creation of new business services (e.g., parts/component regeneration). Offer specialized knowledge. Creation of new product usage patterns.

**Table 5:** Industrial symbiosis drivers and barriers in Śmiłowo Eco-Industrial Park.

Drivers/ Barriers	Level of Occurrence	Type				
		Physical/Technical	Regulatory	Resources	Collaboration	Motivation
Drivers realizing IS exchanges	Intra-organizational	The diverse range of EIP production companies and service activities. SD of transport in IS. Possibilities of the realization of technical challenges in terms of quantity and quality of industrial by-products.	Incentives. Partnership and collaboration with community and local authorities. Promoting the local symbiotic mindset.	Self-organized within Farmutil consortium and bottom-up approach resulting from its strategic goals. Provision of financing for SD projects. Easier direct interaction among Farmutil firms.	Experience of managers from the meat industry sector. Social challenges arise from trusting companies belonging to consortiums to fulfill quantity needs and standards. Flexible production cooperation model based on a supply chain using IS elements.	Expected savings and income. New business opportunities. Competitive advantages. Brand improvement. Farmutil SD strategy. Cost factor influences firms. Increase innovative outreach. Expanding the focus on product and by-product streams.
Drivers relevant to company level	Company	Increasing material interchanges. Take-back systems. Firms cooperate to decrease waste amounts, increase value, and achieve economies in their activity.	Initiative promotion. Use of easily applicable mode for funds. Funds aid the research.	Facilities schemes. Business workshop. Networks with SD data on technical research. The data set of IS methods and procedures. Care for by-products. Models of funds petition.	Knowledge of neighbor firms. Industry organization with good knowledge and sharing. Trust for firms’ collaboration. Integrity by the sustainable use of waste and bio-processes. An easier collaboration promotes a symbiotic mindset.	New business ideas. Good experience leading to more CP activity. Fulfilling company strategy. Clear economic benefits. Industry demand for CE. Possible competitive advantage. Public interest in CE.
	Employee			High-quality employees.	Network expands knowledge.	Professional pride.
Barriers to realizing IS exchanges	Intra-organizational	Delivery and needs correlation. Infrastructural use. Some problems with specific information needs of industrial users concerning waste (classification, distribution, and others).	Uncertainty of local legal regulations and consistency of regional. development policy No enforcement of IS development and appropriate legal requirements.	Unknown profits factor. Various investment processes. Negotiation experience. No skills and resources. Going outside the traditional supply chain requires support. Lack of time and money.	Sharing of companies in costs and profits. Relationship and trust of firms. Needs to improve market and other information flow between companies. Conflict of education and experience results in not seeing IS as a priority.	Limited public awareness of IS. No SD works priority. Scarcity of innovation incentives. No business incentive from the market or regulators. Estimation of financing mechanism viability and lack of business case studies and steering mechanisms.
Barriers relevant to companies	Company	Harder to find buyers than suppliers. Amounts adjustment. Ambient physical area.	Not enough contacts of companies with authorities and municipalities. Decreased explanation of IS. No clear SD tips.	Unknown benefits. Lower raw material cost. Priority of benefits. Problem with employee acquisition. Keeping key competencies. Scarcity of expertise and IS and CE education.	Scale differences. Priority of collaboration. Scarcity of trust. Participation of companies in costs and benefits. Differences in company manner.	Various goals and reasoning. No priority of SD work. IS has low priority due to not being treated as a basic phase of production. Lack of knowledge in the community on SD and CE.
	Employee			No knowledge and perception of the IS principles.	Lack of promoters in companies.	No priority for IS. No employee education.

## Data Availability

All data are presented within the manuscript.

## References

[1] Chertow M.R. (2000). Industrial symbiosis: Literature and taxonomy. Annu. Rev. Energ. Environ..

[2] Frosch R.A., Gallopoulos N.E. (1989). Strategies for manufacturing. Sci. Am..

[3] Neves A., Godina R., Azevedo S.G., Matias J.C.O. (2020). A comprehensive review of industrial symbiosis. J. Clean. Prod..

[4] Friant M.C., Vermeulen W.J.V., Salomone R. (2020). A typology of circular economy discourses: Navigating the diverse visions of a contested paradigm. Resour. Conserv. Recycl..

[5] Cecchin A., Salomone R., Deutz P., Raggi A., Cutaia L., Salomone R., Cecchin A., Deutz P., Raggi A., Cutaia L. (2020). Relating industrial symbiosis and circular economy to the sustainable development debate. Industrial Symbiosis for the Circular Economy. Strategies for Sustainability.

[6] Cecchin A., Salomone R., Deutz P., Raggi A., Cutaia L. (2021). What is in a name? The rising star of the circular economy as a resource-related concept for sustainable development. Circ. Econ. Sust..

[7] Aggeri F. (2021). Industrial eco-parks as drivers of the circular economy. Field Actions Sci. Rep..

[8] Martin M. (2020). Evaluating the environmental performance of producing soil and surfaces through industrial symbiosis. J. Ind. Ecol..

[9] Salomone R., Cecchin A., Deutz P., Raggi A., Cutaia L. (2020). Industrial Symbiosis for the Circular Economy: Operational Experiences, Best Practices and Obstacles to a Collaborative Business Approach.

[10] Shi L., Leal Filho W., Azul A., Brandli L., Özuyar P., Wall T. (2020). Industrial symbiosis: Context and relevance to the sustainable development goals (SDGs). Responsible Consumption and Production. Responsible Consumption and Production.

[11] Ghisellini P., Cialani C., Ulgiati S. (2016). A review on circular economy: The expected transition to a balanced interplay of environmental and economic systems. J. Clean. Prod..

[12] United Nations Environment Programme (UNEP) (2002). Changing Production Patterns: Learning from the Experience of National Cleaner Production Centers.

[13] Clift R., Harrison R.M. (2001). Clean technology and industrial ecology. Pollution: Causes, Effects and Control.

[14] Oldenburg K.U., Geiser K. (1997). Pollution prevention and … or industrial ecology?. J. Clean. Prod..

[15] Liu C., Côté R.P., Zhang K. (2015). Implementing a three-level approach in industrial symbiosis. J. Clean. Prod..

[16] Bocken N.M.P., Short S.W., Rana P., Evans S. (2014). A literature and practice review to develop sustainable business model archetypes. J. Clean. Prod..

[17] Henriques J., Azevedo J., Estrela M., Dias R. (2022). Relating industrial symbiosis and sustainable development goals. Mater. Proc..

[18] Fraccascia L., Giannoccaro I. (2020). What, where, and how measuring industrial symbiosis: A reasoned taxonomy of relevant indicators. Resour. Conserv. Recycl..

[19] Jacobsen N.B. (2006). Industrial symbiosis in Kalundborg, Denmark: A quantitative assessment of economic and environmental aspects. J. Ind. Ecol..

[20] Kosmol L., Maiwald M., Pieper C., Plötz J., Schmidt T. (2021). An indicator-based method supporting assessment and decision-making of potential by-product exchanges in industrial symbiosis. J. Clean. Prod..

[21] Taddeo R. (2016). Local industrial systems towards the eco-industrial parks: The model of the ecologically equipped industrial areas. J. Clean. Prod..

[22] Fraccascia L., Magno M., Albino V. (2016). Business models for industrial symbiosis: A guide for firms. Procedia Environ. Sci. Eng. Manag..

[23] Södergren K., Palm J. (2021). The role of local governments in overcoming barriers to industrial symbiosis. Clean. Environ. Syst..

[24] Poponi S., Arcese G., Pacchera F., Martucci O. (2022). Evaluating the transition to the circular economy in the agri-food sector: Selection of indicators. Resour. Conserv. Recycl..

[25] Dueñas M., García-Estévez I. (2020). Agricultural and food waste: Analysis, characterization and extraction of bioactive compounds and their possible utilization. Foods.

[26] Amicarelli V., Fiore M., Bux C. (2021). Hidden flows assessment in the agri-food sector: Evidence from the Italian beef system. Br. Food J..

[27] Wu J., Guo Y., Li C., Qi H. (2017). The redundancy of an industrial symbiosis network: A case study of a hazardous waste symbiosis network. J. Clean. Prod..

[28] Yedla S., Park H.S. (2017). Eco-industrial networking for sustainable development: Review of issues and development strategies. Clean Technol. Environ. Policy.

[29] Domenech T., Bleischwitz R., Doranova A., Panayotopoulos D., Roman L. (2019). Mapping Industrial Symbiosis Development in Europe-typologies of networks, characteristics, performance and contribution to the Circular Economy. Resour. Conserv. Recycl..

[30] Taddeo R., Simboli A., Morgante A., Erkman S. (2017). The development of industrial symbiosis in existing contexts. Experiences from three Italian clusters. Ecol. Econ..

[31] Kowalski Z., Krupa-Żuczek K. (2007). A model of the meat waste management. Pol. J. Chem. Technol..

[32] Kowalski Z., Makara A. (2021). The circular economy model used in the Polish agro-food consortium: A case study. J. Clean. Prod..

[33] Stokłosa H., Kowalski Z., Makara A. (2019). The use of the circular economy model and cleaner technologies on in the example of the Polish agro-food company Farmutil. Przem. Chem..

[34] Yin R.K. (2011). Applications of Case Study Research.

[35] Kristensen H.S., Mosgaard M.A. (2020). A review of micro level indicators for a circular economy—Moving away from the three dimensions of sustainability?. J. Clean. Prod..

[36] Boons F., Chertow M., Park J., Spekkink W., Shi H. (2017). Industrial symbiosis dynamics and the problem of equivalence: Proposal for a comparative framework. J. Ind. Ecol..

[37] Walker A.M., Vermeulen W.J.V., Simboli A., Raggi A. (2021). Sustainability assessment in circular inter-firm networks: An integrated framework of industrial ecology and circular supply chain management approaches. J. Clean. Prod..

[38] Bicket M., Guilcher S., Hestin M., Hudson C., Razzini P., Tan A., Watkins E. (2014). Scoping Study to Identify Potential Circular Economy Actions, Priority Sectors, Material Flows and Value Chains.

[39] Moraga G., Huysveld S., Mathieux F., Blengini G.A., Alaerts L., Van Acker K., Dewulf J. (2019). Circular economy indicators: What do they measure?. Resour. Conserv. Recycl..

[40] Garrido Azevedo S., Godina R., de Oliveira Matias J.C. (2017). Proposal of a sustainable circular index for manufacturing companies. Resources.

[41] Lehtoranta S., Nissinen A., Mattila T., Melanen M. (2011). Industrial symbiosis and the policy instruments of sustainable consumption and production. J. Clean. Prod..

[42] Pacurariu R.L., Vatca S.D., Lakatos E.S., Bacali L., Vlad M. (2021). A critical review of EU key indicators for the transition to the circular economy. Int. J. Environ. Res. Public Health.

[43] Henriques J., Ferrão P., Castro R., Azevedo J. (2021). Industrial symbiosis: A sectoral analysis on enablers and barriers. Sustainability.

[44] Madsen J.K., Boisen N., Nielsen L.U., Tackmann L.H. (2015). Industrial Symbiosis Exchanges: Developing a Guideline to Companies. Waste Biomass Valorization.

[45] Mortensen L., Kørnøv L. (2019). Critical factors for industrial symbiosis emergence process. J. Clean. Prod..

[46] Makara A., Kowalski Z., Lelek Ł., Kulczycka J. (2019). Comparative analyses of pig farming management systems using the Life Cycle Assessment method. J. Clean. Prod..

[47] (2019). European Circular Economy Stakeholder Platform (ECESP), Kalundborg Symbiosis: Six Decades of a Circular Approach to Production. European Union. https://circulareconomy.europa.eu/platform/en/good-practices/kalundborg-symbiosis-six-decades-circular-approach-production.

[48] European Commission, Regulation (EC) No 1774/2002 of the European Parliament and of the Council of 3 October 2002—Laying down Health Rules Concerning Animal by-Products not Intended for Human Consumption. https://eur-lex.europa.eu/legal-content/EN/TXT/PDF/?uri=CELEX:32002R1774&from=EN.

[49] European Commission, Regulation (EC) No 1069/2009 of the European Parliament and of the Council of 21 October 2009—Laying down health Rules as Regards Animal by-Products and Derived Products not Intended for Human Consumption and Repealing Regulation (EC) No. 1774/2002. https:eur-lex.europa.eu/legal-content/EN/TXT/PDF/?uri=CELEX:32009R1069&from=EN.

[50] Kowalski Z., Kulczycka J., Makara A., Harazin P. (2021). Quantification of material recovery from meat waste incineration–An approach to an updated food waste hierarchy. J. Hazard. Mater..

[51] Makara A., Kowalski Z. (2018). Selection of pig manure management strategies: Case study of Polish farms. J. Clean. Prod..

[52] Tudor T., Adam E., Bates M. (2007). Drivers and limitations for the successful development and functioning of EIPs (eco-industrial parks): A literature review. Ecol. Econ..

